# Metabolic Activity Assessment by 18F-Fluorodeoxyglucose Positron Emission Tomography in Patients after COVID-19 Vaccination

**DOI:** 10.3390/curroncol29020084

**Published:** 2022-02-10

**Authors:** Walid Shalata, Daniel Levin, Janna Fridman, Victoria Makarov, Muhammed Iraqi, Mitchell Golosky, Keren Rouvinov, Waleed Kian, Alexander Yakobson

**Affiliations:** 1 The Legacy Heritage Oncology Center & Dr. Larry Norton Institute, Soroka Medical Center, Ben Gurion University of the Negev, Beer Sheva 84105, Israel; janafr@clalit.org.il (J.F.); kerenro@clalit.org.il (K.R.); waleedkian77@gmail.com (W.K.); alexy@clalit.org.il (A.Y.); 2 Department of Radiology, Soroka Medical Center, Ben Gurion University, Beer Sheva 84105, Israel; DanielLe@clalit.org.il (D.L.); vikam@clalit.org.il (V.M.); 3 The Shraga Segal Department of Microbiology, Immunology, and Genetics, Faculty of Health Science, Ben Gurion University of the Negev, Beer Sheva 8410501, Israel; iraqi@post.bgu.ac.il; 4 Medical School for International Health, Ben Gurion University of the Negev, Beer Sheva 84105, Israel; golosky@post.bgu.ac.il

**Keywords:** coronavirus, COVID-19, vaccine, PET-CT, oncology, hypermetabolic uptake

## Abstract

In the following report, we describe 11 patients with various diagnoses and different treatment statuses (newly diagnosed, receiving treatment, or follow-up) of oncological diseases (breast, lymphoma, melanoma, and head and neck cancers). The patients underwent PET-CT for disease staging or follow-up and it was noted that all patients had areas of hypermetabolic uptake in the axillary lymph-nodes of the ipsilateral upper extremity where the Pfizer-BioNTech coronavirus (COVID-19) vaccine was administered. Following further investigations, including an ultrasound (US), biopsies and an examination of medical records, it was concluded that these findings were the result of the vaccination and not a progression of pre-existing disease.

## 1. Introduction

Cancer is among the leading causes of death globally [1]. In 2018, approximately 18.1 million new cases were discovered, and 9.5 million cancer-related deaths worldwide were reported. In the United States, an estimated 1.8 million new cases of cancer were diagnosed, and at least 600,000 people died from the disease. It is expected that the number of new cancer cases discovered annually will rise to approximately 30 million and the number of cancer-related deaths to 16 million in the next 20 years [1,2].

Cancer screening continues to be a popular, yet controversial issue in the modern medical world [3]. Positron Emission Tomography–Computed Tomography (PET-CT) offers various advantages and represents a highly sensitive modality for cancer screening, in showing the distribution of positron-emitting biomarkers that characterizes tumor metabolic activity [4]. PET-CT is therefore regularly chosen as the preferred imaging modality for staging and follow-up in various types of malignancies [5]. On December 2020, due to the rapid spread of the novel coronavirus (COVID-19), the US Food and Drug Administration (FDA) issued an Emergency Use Authorization (EUA) for the Pfizer-BioNTech COVID-19 vaccine whereby it would be administered in two doses, 21 days apart [6]. Many adverse events were reported after receiving the vaccine, including anaphylaxis, pain at the injection site, tiredness, headache, muscle pain, chills, joint pain, and fever [7,8,9]. Axillary lymph-node enlargement and tenderness are the adverse events most commonly reported after receiving one of the COVID-19 vaccines (either Pfizer-BioNTech or Moderna). In both Pfizer-BioNTech and Moderna vaccines it was noted that this axillary lymph-node enlargement occurred in almost 1.5% of patients after receiving the first dose, and in almost 16% of patients after receiving the second dose. The hypermetabolic uptake that was seen in the PET-CT scan was similar for both vaccine brands [10,11,12,13]. It has been suggested that lymph-node enlargement appears after administration of mRNA vaccines because they are inherently immunostimulatory, with a high immunogenicity compared to other biotechnological vaccines. This also explains the hypermetabolic uptake seen in the axillary region in PET-CT scans [13]. We present a report of false progression of oncological disease in eleven patients that received the Pfizer-BioNTech COVID-19 vaccine and were subsequently followed by PET-CT as a screening modality for their cancer diagnoses.

## 2. Materials and Methods

This was a single institution retrospective, observational study. The study included patients diagnosed with different cancers (of all histologies and stages) who underwent a PET-CT in our hospital between January 2021 and May 2021. Of these, we identified a total of eleven patients who showed metabolic activity following assessment by 18F-fluorodeoxyglucose positron emission tomography. This activity was initially thought to be indicative of disease progression and warranted the need for further investigations. This included a short term follow up (after 2 to 4 months) with an additional PET-CT, an US and, for select patients, a biopsy for further investigation. These assessments along with an examination of the medical records led us to the conclusion that the Pfizer-BioNTech COVID-19 vaccination was responsible for the detected metabolic activity.

## 3. Cases

### 3.1. Case No 1

**Figure 1:**
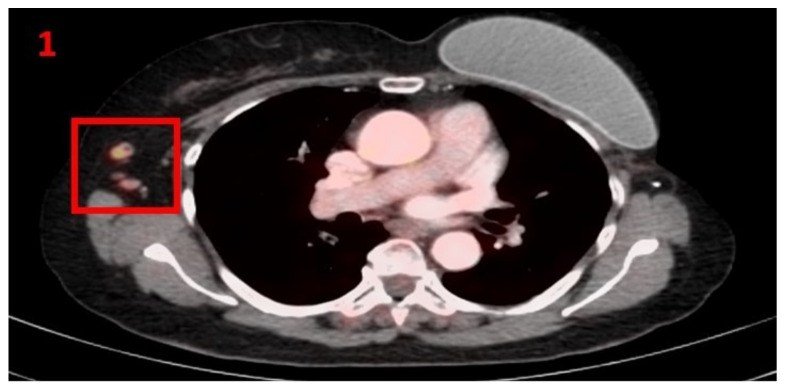
[18F]FDG PET-CT showing hypermetabolic uptake in the 1st patient with lymphadenopathy in the right axillary region (red square).

A 60-year-old female with no family history of cancer was diagnosed in 2005 with invasive ductal carcinoma of the left breast (cT3N0M0), stage IIB, that was estrogen receptor (ER)-negative, progesterone receptor (PR)-positive and human epidermal growth factor receptor 2 (HER2)-negative. She was treated with neoadjuvant chemotherapy of adriamycin (60 mg/m^2^) plus cyclophosphamide (600 mg/m^2^) repeated every 21 days for 4 cycles and followed by weekly taxol (80 mg/m^2^) for 12 cycles. Following neoadjuvant treatment, the patient underwent left radical mastectomy followed by adjuvant radiotherapy and 5 years of tamoxifen. She was followed-up with mammography, US, PET-CT, tumor markers and gynecologic consultations which showed no evidence of malignancy recurrence. In January 2021, she underwent routine screening with [18F]FDG PET-CT that showed foci of hypermetabolic uptake in the right (RT) axillary lymph nodes (LN) (Figure 1). Five days prior to the PET-CT scan, she had received her first Pfizer-BioNTech COVID-19 vaccine in the right arm. The patient was subsequently referred for US of the RT axillary region, which showed LN enlargement with uniform cortical thickening. Examination of the left (LT) axillary region was unremarkable. It was concluded that these were reactive LN. The patient was advised to return for short-term follow-up targeted US or computed tomography (CT) scans of the RT axillary region. Three months later she underwent an US of the bilateral axillary LN area, which did not show any pathological findings.

### 3.2. Case No 2

**Figure 2:**
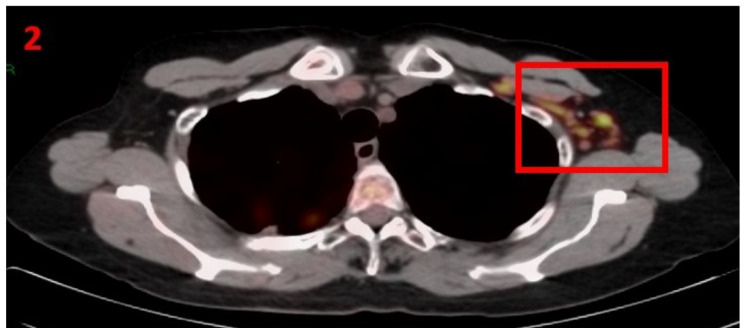
[18F]FDG PET-CT showing hypermetabolic uptake in the 2nd patient with lymphadenopathy in the left axillary region (red square).

A 61-year-old female (BRCA1 carrier) with a family history of BRCA1 breast cancer (mother and grandmother) underwent bilateral preventive (prophylactic) mastectomy in March 2010. Histopathological results showed triple-negative breast cancer in the right breast (pT1N0M0), stage I. The patient was followed-up with US, PET-CT, tumor markers, and gynecologic consultations that showed no evidence of malignancy recurrence. In January 2021, she underwent routine screening with [18F]FDG PET-CT that showed areas of hypermetabolic uptake in the LT axillary LN (Figure 2). Seven days prior to performing her PET-CT scan she received her first Pfizer-BioNTech COVID-19 vaccine in her left arm. The patient was referred for US of the LT axillary region, which showed LN enlargement with uniform cortical thickening. Examination of the RT axillary was unremarkable. It was ultimately concluded that these were reactive LN. The patient was advised to return for short-term follow-up targeted US or CT scans of the LT axillary region. Two months later she underwent an US of the bilateral axillary LN area, which did not show any pathological findings.

### 3.3. Case No 3

**Figure 3:**
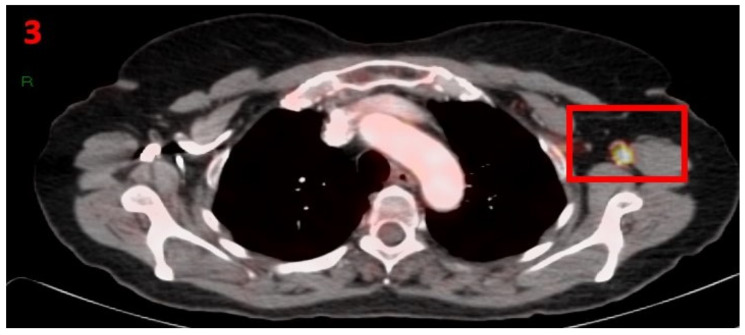
[18F]FDG PET-CT showing hypermetabolic uptake in the 3rd patient with lymphadenopathy in the left axillary region (red square).

A 71-year-old female with no family history of cancer presented in February 2018 with histopathological results showing acral lentiginous melanoma (ALM). PET-CT showed areas of hypermetabolic uptake confirming metastatic disease with lung, abdominal, pelvic and lower limb involvement (cTXN2cM1), stage IV. As the accepted treatment for this metastatic disease, the patient was started on nivolumab (240 mg every 14 days). In January 2021, while still undergoing treatment, she had a [18F]FDG PET-CT for re-staging of her malignancy that showed hypermetabolic uptake only in the LT axillary LN (Figure 3). Thirteen days prior to the PET-CT scan, she received the second Pfizer-BioNTech COVID-19 vaccine in her left arm. The patient was referred for US of the LT axillary region, which showed LN enlargement with uniform cortical thickening. Examination of the RT axillary was unremarkable. It was ultimately concluded that these were reactive LN. The patient was advised to return for short-term follow-up targeted US or CT scans of the LT axillary region to ensure complete resolution. Three months later she underwent [18F]FDG PET-CT, which did not show any pathological findings or hypermetabolic uptake in her axillary LN area.

### 3.4. Case No 4

**Figure 4:**
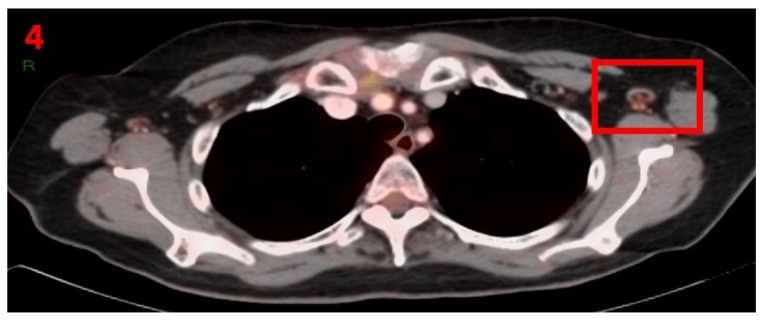
[18F]FDG PET-CT showing hypermetabolic uptake in the 4th patient with lymphadenopathy in the left axillary region (red square).

A 67-year-old female with no family history of cancer presented in December 2020 with a lump measuring 38 × 54 mm in the LT occipital area with regional lymphadenopathy of the LT neck measuring 20 × 10 mm. Head and neck CT showed a calcified lump that did not involve bone, and multiple enlarged lymph nodes in the region of the left neck. The lump was completely excised and the histopathological report showed squamous cell carcinoma of the skin, which was well differentiated (T3N2M0), stage III. In order to rule out metastatic disease, a [18F]FDG PET-CT was performed and showed areas of hypermetabolic uptake in the margins of the excised tissue and in the LT axillary LN (Figure 4). Ten days prior to performing the PET-CT scan the patient received the first Pfizer-BioNTech COVID-19 vaccine in her left arm. No pathological LN were found in the mediastinum or RT axilla. The patient was advised to return for short-term follow-up targeted US or CT scans of the LT axilla to ensure resolution. Three months later she underwent an US, which did not show any pathological findings in her bilateral axillary LN area.

### 3.5. Case No 5

**Figure 5:**
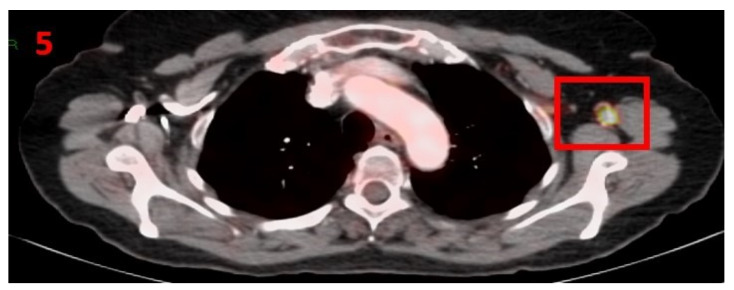
[18F]FDG PET-CT figure showing hypermetabolic uptake in the 5th patient with lymphadenopathy in the left axillary region (red square).

A 61-year-old female with no family history of cancer presented in January 2018 with the chief complaint of melena. She was ultimately diagnosed with poorly differentiated colon adenocarcinoma, which was confirmed with a total body CT. The patient underwent low anterior resection of the sigmoid colon as well as removal of the right ovary. Six of 12 dissected LN were positive for malignancy (T3N2M0), stage IIIC. She was treated appropriately with adjuvant therapy (6 cycles of FOLFOX regimen). In January 2021, a follow-up [18F]FDG PET-CT was performed and showed areas of hypermetabolic uptake in the LT axillary LN (Figure 5). Twelve days prior to the PET-CT scan the patient had received the second Pfizer-BioNTech COVID-19 vaccine in her LT arm. No pathological LN were found in the mediastinum or RT axilla. The patient was advised to return for a short-term follow-up targeted US or CT scan of the LT axilla to ensure resolution. Four months later she underwent [18F]FDG PET-CT, which did not show any pathological findings or hypermetabolic uptake in her axillary LN area.

### 3.6. Case No 6

**Figure 6:**
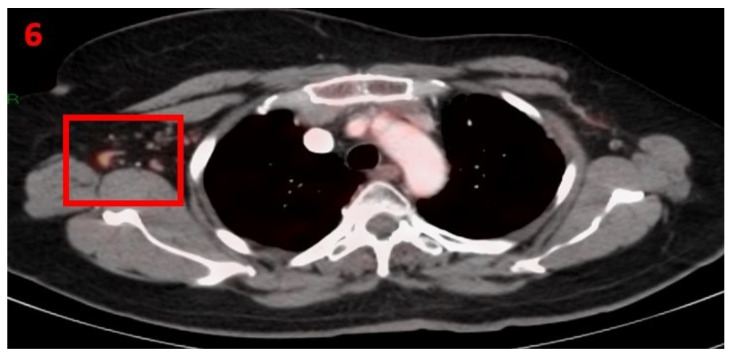
[18F]FDG PET-CT showing hypermetabolic uptake in the 6th patient with lymphadenopathy in the right axillary region (red square).

A 66-year-old female with no family history of cancer, in November 2016, was diagnosed with invasive ductal carcinoma in her LT breast, ER-positive, PR-negative and HER2-positive, with metastasis to the spine (D9 and L3) (cT2N0M1), stage IV. She was treated with weekly taxol (80 mg/m^2^) for 12 cycles, trastuzumab (2 mg/kg) plus pertuzumab (420 mg) repeated every 3 weeks and letrozole daily. During follow-up with mammography, US, PET-CT, tumor markers and gynecologic consultations all showed stable disease. In January 2021, the patient had a routine [18F]FDG PET-CT screening that showed hypermetabolic uptake in the RT axillary LN (Figure 6). Eight days prior to performing her PET-CT scan she had received her second Pfizer-BioNTech COVID-19 vaccine in her RT arm. The patient was advised to return for short-term follow-up targeted US or CT scans of the RT axillary region. Two months later she underwent [18F]FDG PET-CT, which did not show any pathological findings or hypermetabolic uptake in her axillary LN area.

### 3.7. Case No 7

**Figure 7:**
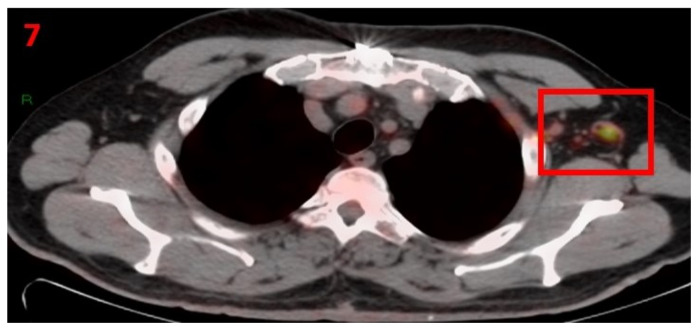
[18F]FDG PET-CT showing hypermetabolic uptake of in 7th patient with lymphadenopathy in the left axillary region (red square).

A 61-year-old male with no family history of cancer was diagnosed with local gastric mucosa-associated lymphoid tissue (MALT) (gastric lymphoma). In August 2013 he was treated with radiotherapy and 8 cycles of retuximab. He achieved radiologic complete response (rCR) and continued to be followed-up with blood tests, CT and PET-CT that showed complete remission. In January 2021, he underwent [18F]FDG PET-CT as part of his routine follow-up screening, which showed hypermetabolic uptake in the LT axillary LN (Figure 7). Six days prior to performing the PET-CT scan he had received the second Pfizer-BioNTech COVID-19 vaccine in his LT arm. The patient was advised to return for short-term follow-up targeted CT or US of the LT axillary region. Two months later he underwent an US, which did not show any pathological findings in his bilateral axillary LN area.

### 3.8. Case No 8

**Figure 8:**
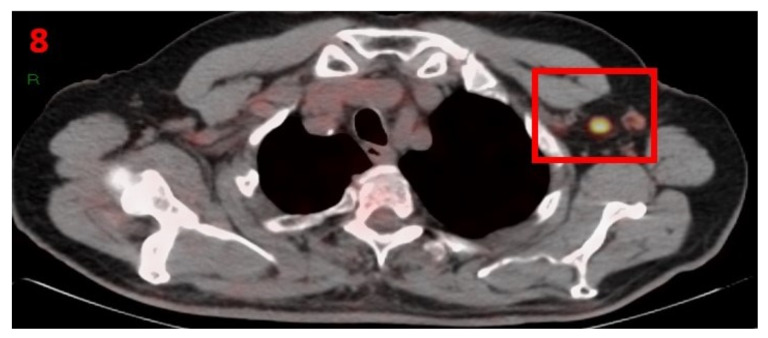
[18F]PSMA PET/CT showing hypermetabolic uptake in the 8th patient with lymphadenopathy in the left axillary region (red square).

A 78-year-old male with no family history of cancer was diagnosed in August 2001 with local prostate adenocarcinoma (Gleason score 6, PSA was 11 ng/mL), for which he underwent radical prostatectomy and his PSA remained undetectable. He was followed-up with routine blood tests, CT and PET- PSMA that showed lack of malignant progression. In February 2021, he underwent a routine PET-PSMA that showed hypermetabolic uptake in the LT axillary region LN (Figure 8). Nine days prior to performing his [18F]PSMA PET/CT scan he received the second Pfizer-BioNTech COVID-19 vaccine in his left arm. The patient was advised to return for short-term follow-up targeted CT or US of the LT axillary region. Three months later he underwent [18F]PSMA PET/CT, which did not show any pathological findings or hypermetabolic uptake in his axillary LN area.

### 3.9. Case No 9

**Figure 9:**
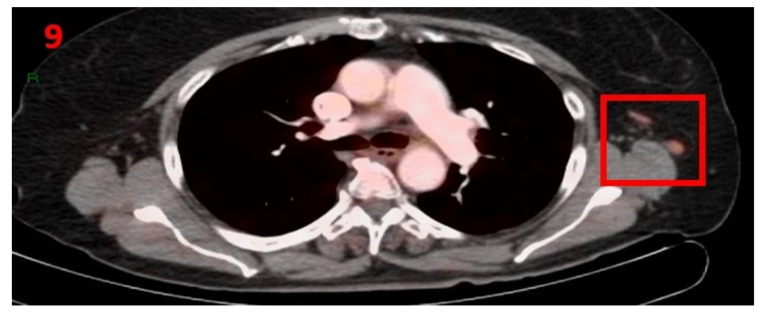
[18F]FDG PET-CT showing hypermetabolic uptake in the 9th patient with lymphadenopathy in the left axillary region (red square).

A 69-year-old female with no family history of cancer was diagnosed with stage 3 follicular lymphoma in December 2016 (liver and inguinal lymphadenopathy). She was treated with 4 cycles of rituximab and reached rCR, and continued to be followed-up with blood tests and PET-CT that showed complete remission. In February 2021, she underwent routine screening with [18F]FDG PET-CT that showed hypermetabolic uptake in the LT axillary LN (Figure 9). Twelve days prior to performing the PET-CT scan she had received the second Pfizer-BioNTech COVID-19 vaccine in her left arm. The patient was advised to return for short-term follow-up targeted CT or US of the LT axillary region. Two months later she underwent an US, which did not show any pathological findings in her bilateral axillary LN area.

### 3.10. Case No 10

**Figure 10:**
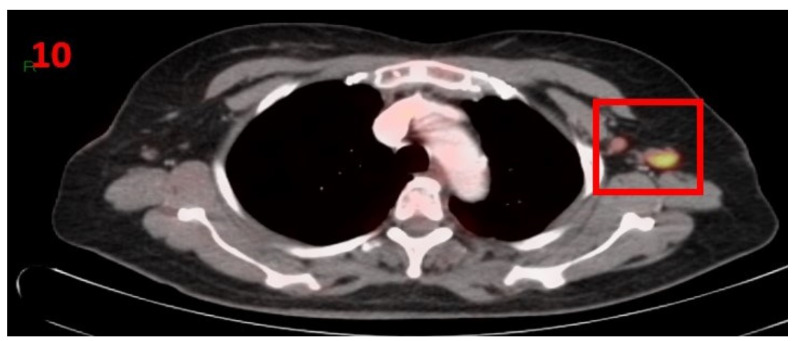
[18F]FDG PET-CT showing hypermetabolic uptake in the 10th patient with lymphadenopathy in the left axillary region (red square).

A 61-year-old female with no family history of cancer was diagnosed in October 2020 with metastatic invasive lobular carcinoma of the RT breast that was ER-positive, PR-positive and HER2-negative (pT1N1M1), stage IV (bone metastasis). In October 2020, she began treatment according to the hormonal receptor status with abemaciclib and letrozole. In February 2021, she underwent routine screening with [18F]FDG PET-CT that showed hypermetabolic uptake only in the LT axillary region LN (Figure 10). Five days prior to performing the PET-CT scan she had received the second Pfizer-BioNTech COVID-19 vaccine in her left arm. The patient was referred for US and biopsy of the LT axilla, which showed LN enlargement with uniform cortical thickening, and the pathology report confirmed no evidence of malignancy. The patient was advised to return for short-term follow-up targeted CT or US of the LT axillary region. Two months later she underwent US, which did not show any pathological findings in the bilateral axillary LN area.

### 3.11. Case No 11

**Figure 11:**
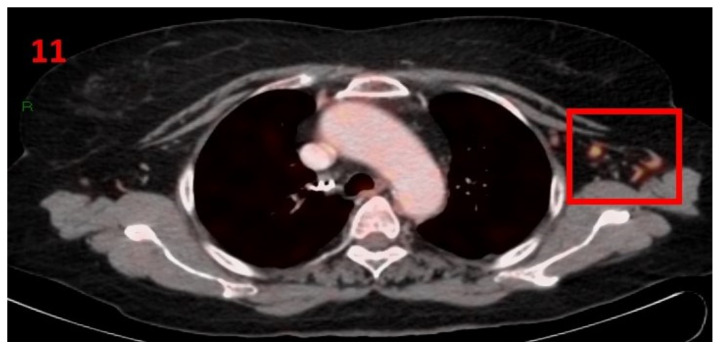
[18F]FDG PET-CT showing hypermetabolic uptake in the 11th patient with lymphadenopathy in the left axillary region (red square).

A 69-year-old female with no family history of cancer was diagnosed with early stage squamous cell carcinoma of the cervix, stage IIB in November 2018 and was treated with combination therapy of cisplatin (30 mg/m^2^) plus 25 fractions (2Gy/fr) of radiotherapy. She continued to be followed-up with blood tests, CT and PET-CT that showed no evidence of malignancy recurrence. In February 2021, she underwent [18F]FDG PET-CT as part of her routine follow-up screening, which showed hypermetabolic uptake in the LT axillary LN (Figure 11). Thirteen days prior to performing the PET-CT scan she had received the second Pfizer-BioNTech COVID-19 vaccine in her left arm. The patient was advised to return for short-term follow-up targeted CT or US of the LT axillary region. Four months later she underwent [18F]FDG PET-CT, which did not show any pathological findings or hypermetabolic uptake in the axillary LN area.

## 4. Discussion

Reactive lymphadenopathy has been found to occur after vaccination administration, more frequently reported in vaccines that especially elicit a relatively strong immune response. [14] The Centers for Disease Control and Prevention (CDC) reported that occurrence of lymphadenopathy was imbalanced, with 58 more cases in the vaccine group than the placebo group (64 vs. 6) in those who received the Pfizer-BioNTech COVID-19 vaccine [9].

We described 11 cases with different oncological diagnoses and severities of disease. All of our patients showed signs on PET-CT of disease recurrence or progression that needed to be confirmed. In order to rule out false positive results, these patients underwent additional imaging and biopsy of the suspected LN. Interestingly, investigations into the patients’ past medical histories revealed a common finding all patients had received the Pfizer-BioNTech COVID-19 vaccine in the 13–5 days preceding the PET-CT (Table 1). Reactive unilateral axillary lymph nodes were demonstrated following vaccination in the ipsilateral upper extremity, as evidenced by unilateral increased uptake of fluorodeoxyglucose (FDG) on PET-CT. This was revealed to be the effect of the vaccine, as opposed to progression of the malignant disease in the region.

Hypermetabolic uptake on PET-CT and axillary lymphadenopathy should be carefully investigated for possible progression of oncologic disease. It is known that activated inflammatory cells increase their expression of glucose transporters and have markedly increased breakdown of glucose, resulting in high FDG uptake [15]. Moreover, during periods of infection, active fibrosis, and active granulomatous processes such as sarcoidosis, there is hypermetabolic uptake of FDG that often results in false-positive PET-CT scans for malignancy [16]. Since the initiation of the global COVID-19 vaccination programs, multiple studies have cited the occurrence of hypermetabolic axillary lymph nodes in patients after receiving the COVID-19 vaccine, which has complicated accurate diagnosis [11,17,18]. A meta-analysis including over 2000 patients undergoing 2-[18F]-FDG PET/CT revealed hyper-metabolic axillary lymph nodes with a prevalence of 37% after COVID-19 vaccination [19]. With such frequencies it is vital that medical staff and patients around the world be aware of such COVID-19 vaccine-related immune responses and the resulting diagnostic complications, not only to prevent potential misdiagnoses and vital treatment delays but also to spare patients any unnecessary additional testing and anxiety. As mentioned earlier, axillary LN enlargement and hypermetabolic uptake were noted as an adverse event (AE) after administration of both Pfizer-BioNTech and Moderna vaccines. Furthermore, Schroeder et al. reported in their study the admission of two nuclear medicine physicians, independently, who reviewed the images and were blinded to injection laterality and the number of days since vaccination. It was noted that these AE were reported in both Pfizer-BioNTech and Moderna vaccines in patients who underwent 11c-choline PET CT (23.1% of patients) and [18F] FDG PET-CT (7.4% of patients) examinations [10,11,20]. Only a single case reported the same AE for a female with history of Classic Hodgkin Lymphoma who underwent follow-up [18F] FDG PET-CT, after administration of adenovirus-vectored Oxford-AstraZeneca COVID-19 vaccine. **[21]** We hypothesize that the reason for more events that reported after the administration of both Pfizer-BioNTech and Moderna vaccines was the use of these vaccines was more common than that of other vaccines.

## 5. Conclusions

Although the differential diagnosis of unilateral axillary lymphadenopathy is extensive, we suggest that a thorough vaccination history be obtained, as vaccination status provides a possible benign etiology. Furthermore, we aim to raise awareness of the difficulty in detecting malignancy recurrence as COVID-19 vaccine administration becomes more prevalent. To avoid unnecessary diagnostic dilemmas, treatment delays, additional PET-CT scans and patient anxiety, a time-controlled protocol is recommended. Our recommendation includes that recently vaccinated cancer patients are subjected to follow-up imaging shortly after administration of their second vaccine dose. Successful execution of this would require stringent coordination between clinicians, nurses and the patient, but would ensure complete imaging resolution and limit the number of false-positive biopsy referrals. Optimization of the exact time-lapse between the second vaccination and the short-term imaging would occur as more patients are subjected to this new protocol. By performing post-vaccine imaging and ensuring that diagnostic imaging is performed in a time frame that minimizes the risk of hypermetabolic axillary lymph node interference, the risk of the aforementioned challenges would be significantly reduced.

## Figures and Tables

**Table 1:** Patient Details.

Patient	Primary Tumor	Vaccination Location	Period between Vaccination and PET-CT	PET-CT Result	Number of Vaccines Received	Diameter of the LN	SUV of LN	Type of Vaccine
Case no.1	Breast cancer	Right arm	5 days	Hypermetabolic uptake in the right axillary region and lymphadenopathy	First vaccine	10 mm	4.6	Pfizer-BioNTech
Case no.2	Breast cancer	Left arm	7 days	Hypermetabolic uptake in the left axillary region and lymphadenopathy	First vaccine	7 mm	3.7	Pfizer-BioNTech
Case no.3	Melanoma	Left arm	13 days	Hypermetabolic uptake in the left axillary region and lymphadenopathy	Second vaccine	5 mm	1	Pfizer-BioNTech
Case no.4	Scc of skin	Left arm	10 days	Hypermetabolic uptake in the left axillary region and lymphadenopathy	First vaccine	9 mm	4.2	Pfizer-BioNTech
Case no.5	Colon cancer	Left arm	12 days	Hypermetabolic uptake in the left axillary region and lymphadenopathy	Second vaccine	8 mm	6	Pfizer-BioNTech
Case no.6	Breast cancer	Right arm	8 days	Hypermetabolic uptake in the right axillary region and lymphadenopathy	Second vaccine	10 mm	3	Pfizer-BioNTech
Case no.7	Gastric lymphoma	Left arm	6 days	Hypermetabolic uptake in the left axillary region and lymphadenopathy	Second vaccine	10 mm	3	Pfizer-BioNTech
Case no.8	Prostate cancer	Left arm	9 days	Hypermetabolic uptake in the left axillary region and lymphadenopathy	Second vaccine	8 mm	5	Pfizer-BioNTech
Case no.9	Follicular lymphoma	Left arm	12 days	Hypermetabolic uptake in the left axillary region and lymphadenopathy	Second vaccine	7 mm	1.9	Pfizer-BioNTech
Case no.10	Breast cancer	Left arm	5 days	Hypermetabolic uptake in the left axillary region and lymphadenopathy	Second vaccine	11 mm	4.5	Pfizer-BioNTech
Case no.11	Uterine cervix	Left arm	13 days	Hypermetabolic uptake in the left axillary region and lymphadenopathy	Second vaccine	9 mm	2.1	Pfizer-BioNTech

No, number; PET-CT, Positron emission tomography–computed tomography; LN, lymph nodes; SUV, standardized uptake values; Scc, squamous cell carcinoma; mm, millimeter.

## Data Availability

The data presented in this study is available in this article.
